# Comparative transcriptome profiles of four sexually size dimorphic fish

**DOI:** 10.1038/s41597-022-01887-1

**Published:** 2022-12-17

**Authors:** Li-Fei Luo, Zi-Sheng Xu, Dan-Yang Li, Zhen Hu, Ze-Xia Gao

**Affiliations:** 1 College of Fisheries, Key Lab of Freshwater Animal Breeding, Ministry of Agriculture/Key Lab of Agricultural Animal Genetics, Breeding and Reproduction of Ministry of Education/Engineering Research Center of Green development for Conventional Aquatic Biological Industry in the Yangtze River Economic Belt, Ministry of Education, Huazhong Agricultural University, Wuhan, 430070 China; 2 Hubei Hongshan Laboratory, Wuhan, 430070 China; 3 Hubei Aquatic Products Technology Promotion Station, Wuhan, 430060 China; 4 Engineering Technology Research Center for Fish Breeding and Culture in Hubei Province, Wuhan, 430070 China

**Keywords:** Development, Transcriptomics

## Abstract

Sexual size dimorphism is widespread in fish species. Although sex growth differences in multiple species have been studied successively, the commonalities of regulatory mechanisms across sexually dimorphic species are unknown. In this study, we performed RNA-seq analysis of four representative fish (loach, half-smooth tongue sole, yellow catfish, and Nile tilapia) with significant growth differences between females and males. Clean reads were identified from four fish species, ranging from 45,718,052 to 57,733,120. Following comparison transcriptome analysis, there were 1,132 and 1,108, 1,290 and 1,102, 4,732 and 4,266, 748 and 192 differentially expressed genes (DEGs) in the brain and muscle of loach, half-smooth tongue sole, yellow catfish, and Nile tilapia, respectively. Furthermore, the expression levels were validated by quantitative real-time PCR (qRT-PCR). Comparative transcriptome profiles of four fish described here will provide fundamental information for further studies on the commonalities of sexually size dimorphic fish in regulating growth differences between females and males.

## Abstract (summary)

**Table:** 

Measurement(s)	Transcripts expression profile
Technology Type(s)	Illumina HiSeq
Factor Type(s)	sex
Sample Characteristic - Organism	Misgurnus anguillicaudatus • Cynoglossus semilaevis • Pelteobagrus fulvidraco • Oreochromis niloticus

## Background & Summary

Many fish species display sexual dimorphism, the most common of which is sexual size dimorphism, wherein one sex is larger than the other, but exhibits species specificity. For example, common carp (*Cyprinus carpio*), rainbow trout (*Oncorhynchus mykiss*), Japanese flyuounder (*Paralichthys olivaceus*), and half-smooth tongue sole (*Cynoglossus semilaevis*) show the most extreme sexual size dimorphism with females being larger and growing faster than males^1–3^. In contrast, in some species, such as Nile tilapia (*Oreochromis niloticus*), yellow catfish (*Pelteobagrus fulvidraco*), and channel catfish (*Ictalurus punctatus*), the growth rate and body size of males is faster and larger than females^4–6^. The growth of vertebrates is regulated by growth hormones/insulin-like growth factors secreted by the hypothalamic-pituitary-gonad (HPG) axis and other tissues^7^. So many studies on the sexual size dimorphism in fish mainly have focused on the genes and hormones related to the HPG axis^8–10^. As we all know, most complex traits are controlled by multiple genes, so studies that focus on one or two genes and hormones related to the HPG axis cannot fully reveal the regulatory mechanism of sexual size dimorphism. Therefore, the molecular mechanism of sexual size dimorphism in fish is still unclear.

The difference in gene expression between sexes is thought to be the key contributor to the manifested phenotypic differences^11,12^. Therefore, in order to understand the causes of sexual size dimorphism, numerous studies have focused on differentially expressed genes (DEGs) between females and males. Comparative transcriptome analysis can help people find DEGs at the entire genome level, and has been widely applied to study sex-biased genes in animals including fish^13,14^, swimming crab^15^, pig^16^, cattle^17^, and chicken^18^. However, the existing transcriptome mostly focus on expression pattern of one or several specific organs at a single specie level. The degree to which sex-biased expression is conserved across the specie lineage and the extent of conservation in different tissues and organ systems are unknown. Assessing the expression of sex-biased genes across species will contribute to a comprehensive understanding of the molecular mechanisms regulating phenotypic sex differences^19^. So far, no studies comprehensively and accurately elucidate how gene expression differs between the sexes in a broad range of sexual size dimorphic fish species and tissues, and the universality of the regulation mechanism of growth differences between females and males in sex-dimorphic fish is still unclear.

Loach (*Misgurnus anguillicaudatus*), half-smooth tongue sole (*Cynoglossus semilaevis*), yellow catfish (*Pelteobagrus fulvidraco*), and Nile tilapia (*Oreochromis niloticus*) are all important economic fish in aquaculture^20–23^. However, they all have sexually dimorphic growth patterns with significant growth differences between female and male individuals. *M. anguillicaudatus* and *C. semilaevis* have growth advantage in females while *P. fulvidraco* and *O. niloticus* have growth advantage in males, which affects the fish yield and economic value of these cultured species^24^. It is not clear whether there are some commonalities in the molecular mechanisms regulating the growth differences between females and males in sex-growth dimorphic fish (including fish with both female and male growth dominance). Therefore, exploring the common regulation genes and pathways between females and males of these four representative fish will help us further understand the molecular mechanism of sexual size dimorphism in fish, thus providing an important theoretical basis for breeding fast growing and uniform varieties in aquaculture.

**Fig. 1:**
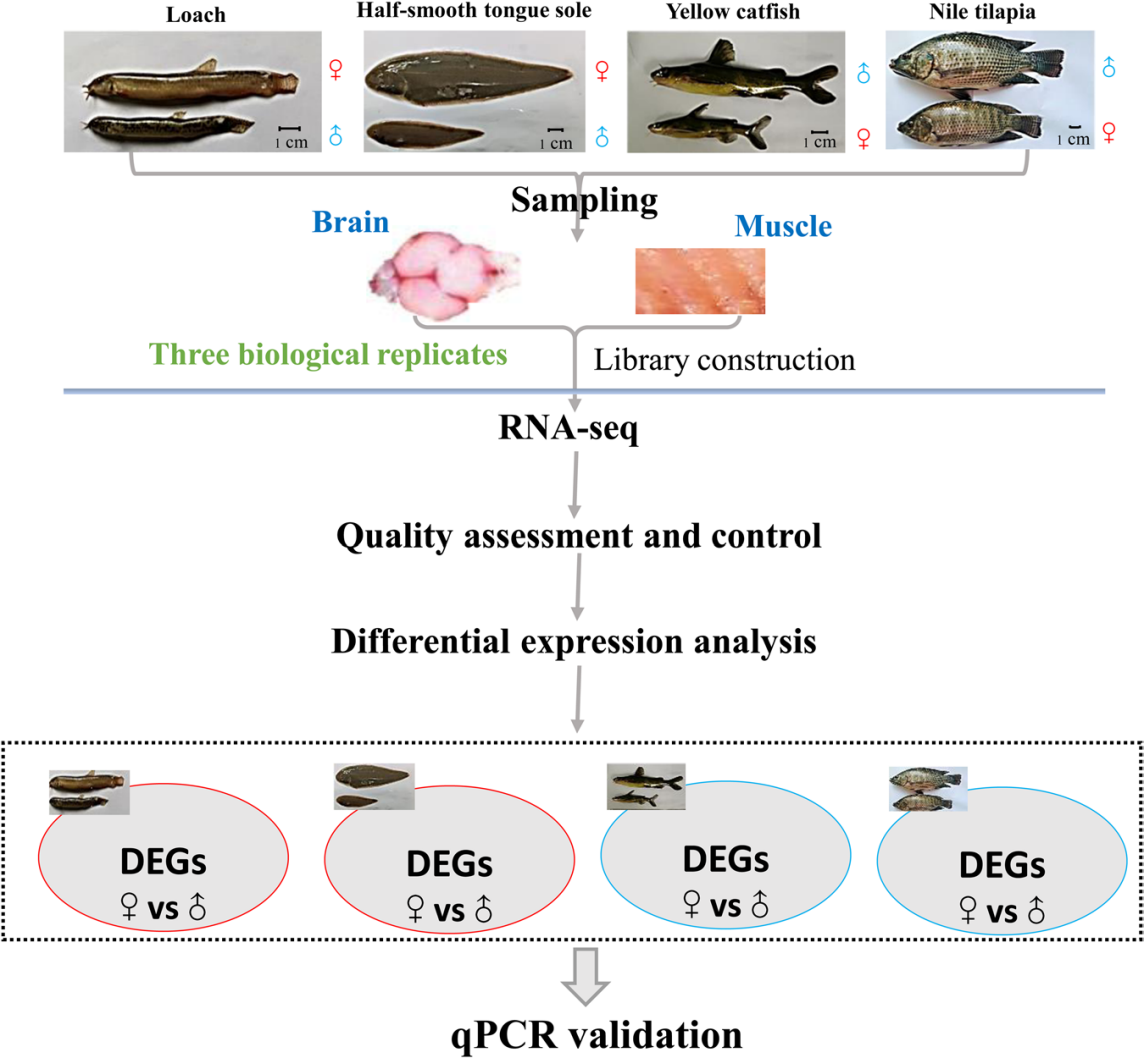
Flow chart of comparative transcriptome analysis.

In this study, we performed transcriptomic sequencing on tissues of four representative fish (loach, half-smooth tongue sole, yellow catfish, and Nile tilapia) during significant growth differences between females and males by RNA-seq, including 2 tissues (brain and muscle), and 48 libraries (three biological replicates in each sample). Quality control was conducted to evaluate the quality of our transcriptome data using FastQC, and a high-quality dataset is presented. Additionally, we performed comparative transcriptomic analyses of four sexually size dimorphic fish with the aim of identifying the DEGs between females and males in four species. The schematic overview of the study design or workflow is shown in Fig. 1. Our work represents a valuable resource for re-use, and will provide fundamental information for further studies on the commonalities of sexually size dimorphic fish in regulating growth differences between females and males.

## Methods

### Ethic statement

All experiments were conducted in accordance with the guidelines of the National Institute of Health Guide for the Care and Use of Laboratory Animals and approved by the Research Ethics Committee, Huazhong Agricultural University, Wuhan, China (approval ID: SYXK2015-0084). All surgery was performed under MS-222 (Sigma, Saint Louis, MO, USA; 100 mg/L) anesthesia, and all efforts were made to minimize suffering.

### Experimental fish and sample collection

**Fig. 2:**
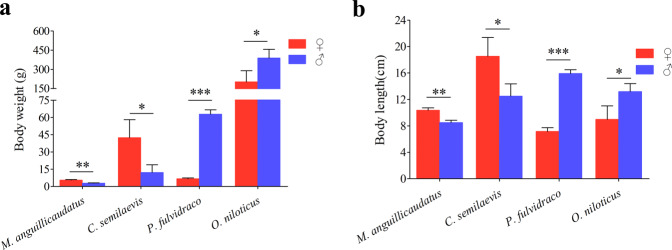
Comparison of body length and body weight between females and males of four representative fish. T-test was used to calculate significant levels between the indicated groups. *P < 0.05, **P < 0.005, and ***P < 0.0001.

*M. anguillicaudatus* used in the experiment was collected from the artificial breeding population of our laboratory, and *C. semilaevis* was purchased from Qingdao, Shandong Province. *P. fulvidraco* and *O. niloticus* were provided by Taishan Base of Research Center of Haida Group and Wuxi Fisheries College of Nanjing Agricultural University, respectively. Each fish species was selected from a full sibling family. Body weight and length were measured and compared by student’s t-test. The average weight of females and males of *M. anguillicaudatus* was 5.42 ± 0.66 g and 2.77 ± 0.36 g, respectively, which indicated that the weight of females was 95.67% heavier than those of males (*P < *0.005). In *C. semilaevis*, the average weight of females and males was 42.43 ± 15.65 g and 12.06 ± 6.80 g, respectively, which indicated that the weight of females was 3.5 times heavier than those of males (*P < *0.05) (Fig. 2a). The comparisons of body length between females and males in these two species also showed that females grew faster than males (Fig. 2b). However, in *P. fulvidraco* and *O. niloticus*, males grew faster than females, especially in *P. fulvidraco*, the difference between females and males was obvious (*P < *0.0001), with males 9.57 times heavier and 2.23 times longer than females. Nine healthy females and males from each species were selected for transcriptome sampling, and three biological replicates were set up, each containing three fish. After anesthetizing the fish with 100 mg/L MS-222, brain and muscle tissues were quickly dissected, frozen in liquid nitrogen and stored at −80 °C for subsequent RNA extraction.

### Libraries construction and sequencing

Total RNA was extracted from each sample by trizol with manufacturer’s protocol (Ambion). RNA integrity assessment was conducted by Agilent 2100 Bioanalyzer (Agilent Technologies). The procedures of RNA purification and libraries construction were performed by using TruSeq Stranded mRNA LTSample Prep Kit (Illumina) following the manufacturer’s instructions. Briefly, high quality mRNA was used to synthesize double stranded cDNA and purified by AMPure XP beads (Beckman Coulter, Beverly, USA). After that, the purified cDNA fragments were repaired on the 3′ end and adenylated before being ligated to sequencing adapters. Subsequently, the libraries were purified, enriched by PCR, and purified again to generate the final libraries. At last, the libraries were sequenced on the Illumina sequencing platform (HiSeqTM 2500), generating 150 bp paired-end reads.

### Transcriptome assembly and gene expression level analysis

Quality control and reads statistics were determined by Trimmomatic^25^_._ At the same time, Q30 and GC contents of the clean reads were calculated, and all the downstream analyses were based on clean reads with high quality. The high-quality clean data of *M. anguillicaudatus* were then de novo assembled using Trinity software with min_kmer_cov set to 2 by default and all other parameters set default^26^, then the transcriptome assembly was then assessed using BUSCO (Benchmarking Universal Single-Copy Orthologs). And the final clean reads of *C. semilaevis*, *P. fulvidraco*, and *O. niloticus* were mapped to the corresponding reference genome using hisat2^27^. The expression level of each transcript was calculated by the expected number of Fragments Per Kilobase of transcript sequence per Millions base pairs sequenced (FPKM) method^28^, and the FPKM value and the read counts of each gene was calculated by cufflinks and htseq-count^29^, respectively.

### Differential expression analysis

To identify the differential expression genes (DEGs) in the brain and muscle tissues of each species, DESeq R package with estimate Size Factors and nbinom Test was performed to quantify the expression of two expression profiles^30^. The unigenes with *P*-value < 0.05 and |log2(fold-change)| >1 were identified as significant DEGs. In the comparisons of brain, there were 1,132, 1,290, 4,732, and 748 DEGs in loach, half-smooth tongue sole, yellow catfish, and Nile tilapia, respectively. In the comparisons of muscle, 1,108, 1,102, 4,266, and 192 DEGs were identified separately in those four species. The information of DEGs of four fish are available on Figshare.

### Confirmation by quantitative real-time PCR (qRT-PCR)

In order to validate the results of RNA-seq, several DEGs in brain and muscle tissues of four fishes were selected for quantitative real-time polymerase chain reaction (qRT-PCR) analysis. The total RNA of the brain and muscle tissues from both sexes of *M. anguillicaudatus*, *C. semilaevis*, *P. fulvidraco*, and *O. niloticus* was reverse transcribed by using the PrimeScriptRT reagent Kit (Takara) following the manufacturer’s protocol. Primers were designed using Primer Premier 5.0 software and were listed in Supplementary Table 1. Each 20 μL reaction volume contained 10 uL 2 × Hieff^®^ qPCR SYBR Green Master Mix (Yeasen, Shanghai, China), 1.0 uL diluted cDNA template, 0.8 uL each of sense and reverse primers, and 7.4 uL ddH_2_O. The qRT-PCR reaction was performed using the Applied Biosystems QuantStudio 6 Flex Real-time PCR System (Applied Biosystems, Foster City, CA, USA) with the following program: 95 °C for 30 s, followed by 40 cycles of 95 °C for 5 s, 60 °C for 30 s and 72 °C for 30 s. Five biological replicates were performed in each reaction. The expression of *β-actin* was served as the reference for internal standardization to normalize the Ct values to conduct the 2^−ΔΔCt^ method^31^.

## Data Records

All sequencing data of *M. anguillicaudatus*, *C. semilaevis*, *P. fulvidraco*, and *O. niloticus* were uploaded to the Sequence Read Archive (SRA) of the National Center for Biotechnology Information under accession number SRP313711, SRP313744, SRP314481, and SRP313936^32–35^, respectively. The information of annotations for unigenes of *M. anguillicaudatus*, and the information of DEGs of four fish can be found on the Figshare^36^.

## Technical Validation

**Fig. 3:**
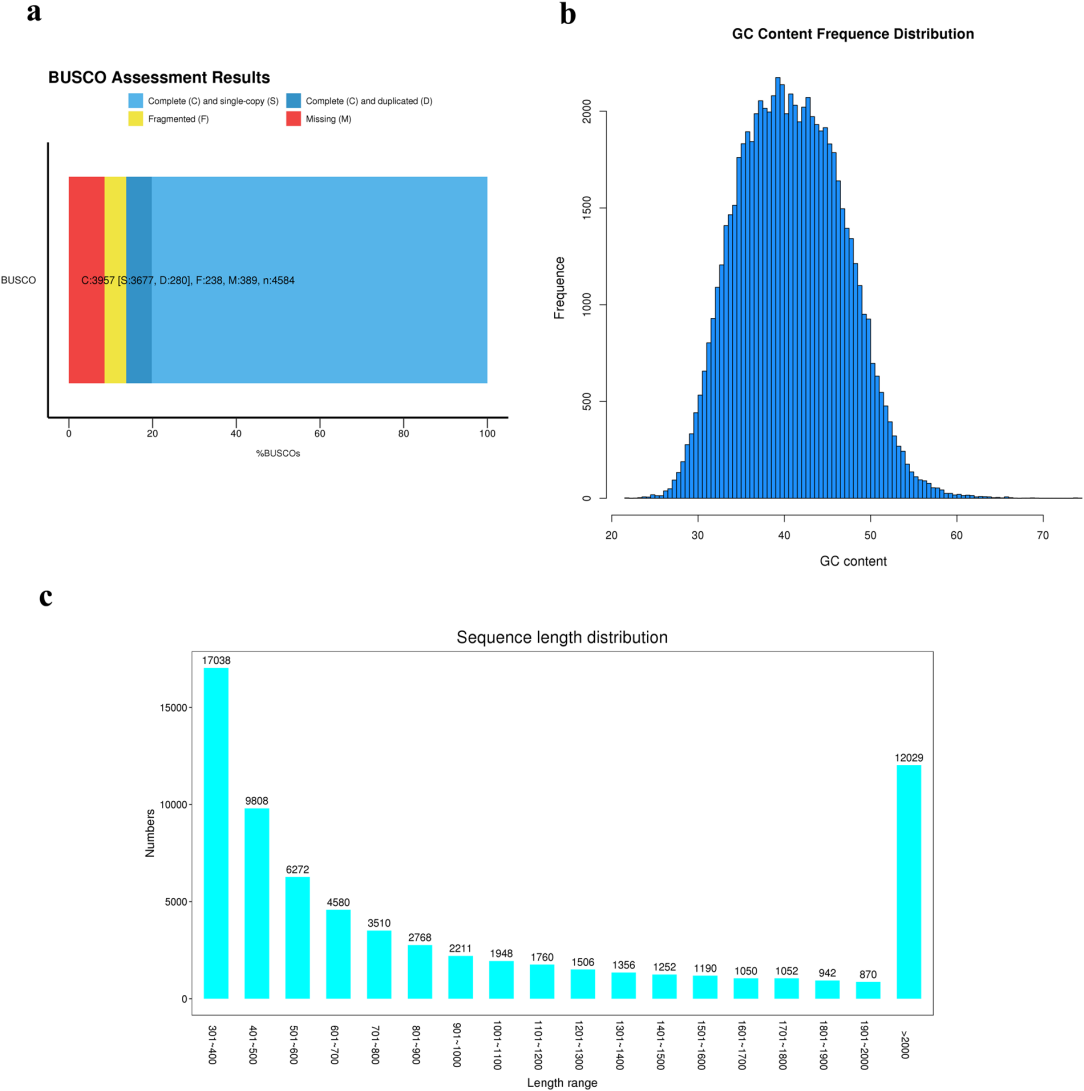
Quality assessment of *M. anguillicaudatus* de novo assembled data. (**a**) BUSCO assessment results of *M. anguillicaudatus* assembled data. Complete and single-copy BUSCOs (S): the number of genes that are completely matched and match the previous one in BUSCO library; Complete and duplicated BUSCOs (D): number of duplicated and duplicated genes in the BUSCO library; Fragmented BUSCOs (F): number of genes in partially matched BUSCO library; Missing BUSCOs (M): number of unmatched genes in the BUSCO library; Total BUSCO groups searched (n): the chosen BUSCO library of all the number of genes. (**b**) GC content distribution map of unigenes. (**c**) Length distribution of unigenes.

**Fig. 4:**
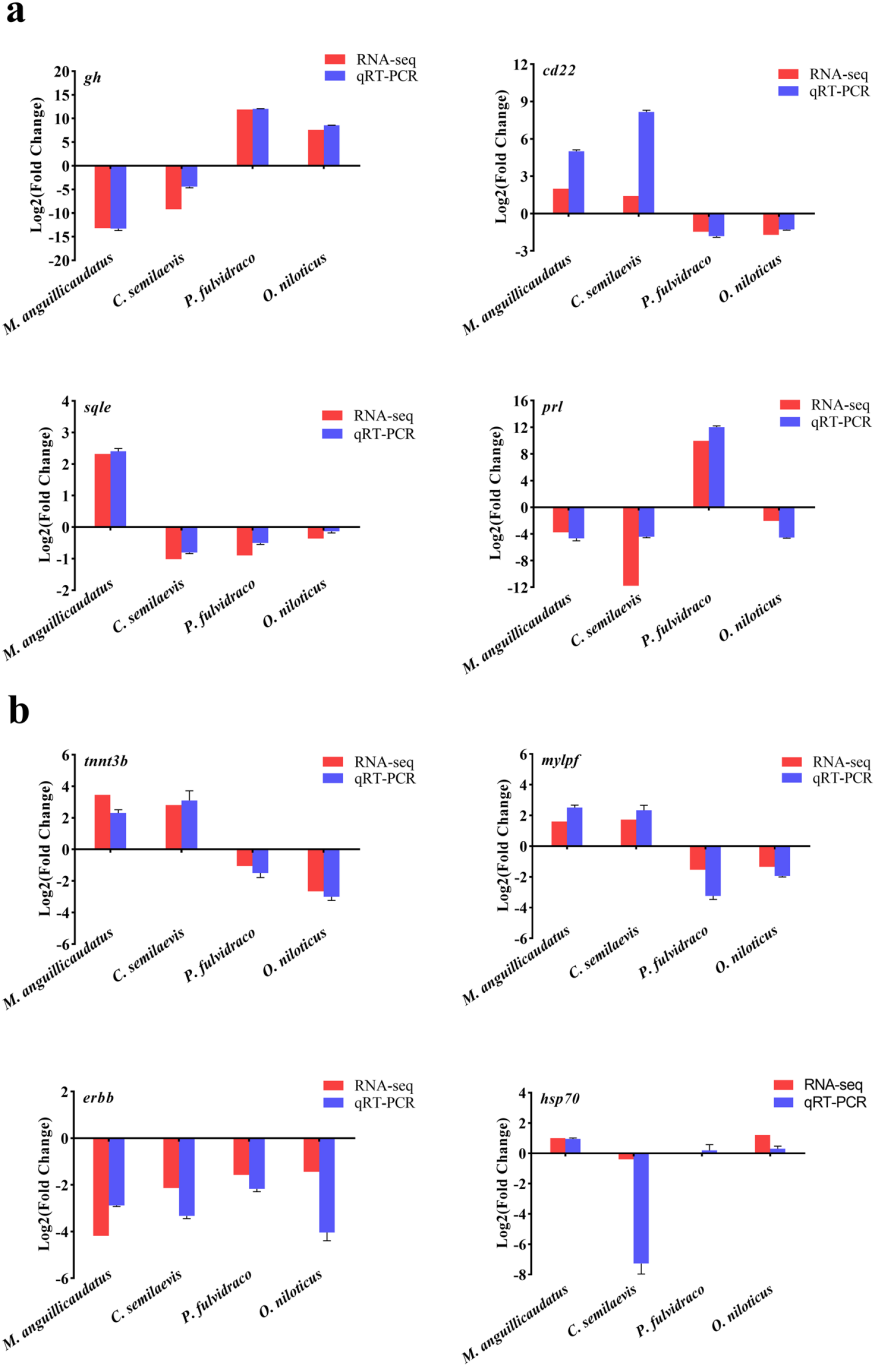
Illustrating of qRT-PCR confirmation for RNA-seq. Each bar represents the expression fold change in a gene compared to that in the males. (**a**) Expression patterns of DEGs in four fish brains. (**b**) Expression patterns of DEGs in four fish muscles.

A similar number of raw reads was obtained for four fish species, ranging from 46,481,320 to 60,661,760 (Supplementary Table 2). After trimming, a total of 45,718,052 to 57,733,120 clean reads remained and the overall mapping efficiency of these reads against the reference genome of the corresponding species ranged from 88.43 to 97.89%, the above results indicated that the quality of the sequencing data was high enough for subsequent analysis. BUSCO analysis of *M. anguillicaudatus* de novo assembled data revealed that a total of 86.32% genes that were completely matched in BUSCO library (3,957 out of 4,584 genes) (Fig. 3a). Among them, 80.27% were those of complete and single-copy BUSCOs (3,677 out of 4,584) and 6.11% were complete and duplicated BUSCOs (280 out of 4,584). Although the proportion of complete alignments is not as high as that of the previous loach transcript assembly, the proportion of complete and single-copy BUSCOs is higher than the previous 70.6%^37^. Distribution map of GC content and length of unigenes in *M. anguillicaudatu* were shown in Fig. 3b,c. Collectly, it indicated high quality of loach transcriptome assembly. As for species with reference genomes (half-smooth tongue sole, yellow catfish, and Nile tilapia), after all the clean reads were mapped onto the corresponding reference genome, the number and percentage of uniquely mapped reads and multiply mapped reads was calculated and presented in Table 1. The correlation of gene expression levels between samples is an important index to verify the reliability of an experiment, and the Pearson correlation coefficient (r) with a square value greater than 0.85 was a prerequisite for differential expression analysis (Supplementary Fig. 1 and Fig. 2). Additionally, although the values of the log2(fold-change) from the transcriptomic analysis and qRT-PCR analysis were different, the differential expression levels of these selected genes by qRT-PCR were highly consistent with those observed by RNA-seq (Fig. 4).

**Table 1:** Statistics analysis of clean reads mapping onto reference genome.

Species	Sample name	Number of uniquely mapped reads	Percentage of uniquely mapped reads%	Number of multiply mapped reads	Percentage of multiply mapped reads%
*C. semilaevis*	F_brain1	45,080,563	91.28%	1,275,758	2.58%
	F_brain2	47,268,898	90.83%	1,399,890	2.69%
	F_brain3	43,645,440	90.99%	1,289,586	2.69%
	F_muscle1	41,421,038	86.18%	3,677,788	7.65%
	F_muscle2	47,897,954	86.65%	4,300,193	7.78%
	F_muscle3	45,414,577	87.15%	3,866,463	7.42%
	M_brain1	45,505,320	91.43%	1,254,380	2.52%
	M_brain2	47,031,880	91.50%	1,246,549	2.43%
	M_brain3	47,045,429	91.65%	1,315,726	2.56%
	M_muscle1	49,216,376	87.87%	3,659,373	6.53%
	M_muscle2	39,491,270	87.32%	3,022,780	6.68%
	M_muscle3	48,628,744	87.02%	3,807,886	6.81%
*P. fulvidraco*	F_brain1	45,394,150	92.63%	1,299,059	2.65%
	F_brain2	47,837,486	92.47%	1,268,419	2.45%
	F_brain3	46,165,503	92.26%	1,268,917	2.54%
	F_muscle1	47,219,749	86.65%	5,460,284	10.02%
	F_muscle2	44,567,138	88.57%	3,987,067	7.92%
	F_muscle3	45,011,306	86.83%	5,024,160	9.69%
	M_brain1	51,683,137	92.64%	1,363,262	2.44%
	M_brain2	50,720,918	92.38%	1,381,930	2.52%
	M_brain3	47,488,856	92.62%	1,272,313	2.48%
	M_muscle1	49,184,986	87.24%	4,942,037	8.77%
	M_muscle2	46,620,994	85.13%	6,241,636	11.40%
	M_muscle3	46,092,038	87.05%	5,237,187	9.89%
*O. niloticus*	F_brain1	46,561,189	91.50%	1,123,021	2.21%
	F_brain2	44,809,941	92.44%	1,045,914	2.16%
	F_brain3	47,442,736	90.26%	1,281,658	2.44%
	F_muscle1	43,722,771	87.46%	4,793,153	9.59%
	F_muscle2	43,776,082	89.96%	3,877,328	7.97%
	F_muscle3	39,746,755	87.13%	4,296,799	9.42%
	M_brain1	47,446,250	91.92%	1,157,992	2.24%
	M_brain2	46,747,697	90.57%	1,174,645	2.28%
	M_brain3	46,200,304	93.66%	1,145,974	2.32%
	M_muscle1	43,873,255	87.36%	4,070,023	8.10%
	M_muscle2	46,863,229	88.37%	4,204,486	7.93%
	M_muscle3	48,019,110	90.35%	4,005,195	7.54%

Taken together, our findings present a high-quality transcriptomic dataset characterizing differences in transcription levels between females and males in sexually size dimorphic fish, benefiting the study of exploring the common genes regulating the growth differences between females and males in fish with sexually size dimorphism.

## Supplementary information

Supplementary information

## Data Availability

All software used in this study were executed according to the manual and protocols of the published bioinformatic tools. FastQC, version 0.11.3, was used for the quality check of the raw FASTQ sequencing files. https://www.bioinformatics.babraham.ac.uk/projects/fastqc/. The versions and parameters of the transcriptome assembly and expression analysis software described in the methods section are as follows: Trimmomatic, version 0.36, LEADING:3 TRAILING:3 SLIDINGWINDOW:4:15 MINLEN:50. Trinity, version 2.4.0, --seqType fq--SS_lib_type RF. hisat2, version 2.2.1.0, --rna-strandness rf –fr. cufflinks, version 2.2.1, --library-type fr-firststrand. htseq-count, version 0.9.1, -s reverse DESeq, version 1.18.0, pvalue < 0.05, |log2FoldChange| >1
