# Supplementary material for: Comparative transcriptome profiles of four sexually size dimorphic fish
Source: Sci Data. 2022 Dec 17;9:774. doi: 10.1038/s41597-022-01887-1 (PMC9759545; doi:10.1038/s41597-022-01887-1)
Supplement: Supplementary file 1 — Supplementary information [file 41597_2022_1887_MOESM1_ESM.pdf]

Supplemental Materials

Comparative transcriptome profiles of four sexually size dimorphic fish

Li-Fei Luo, Zi-Sheng Xu, Dan-Yang Li, Zhen Hu, Ze-Xia Gao

Supplementary Materials Index

Supplementary Fig. 1. Heatmap coefficient matrix between samples of *M. anguillicaudatus* and *C. semilaevis*. Page 2

Supplementary Fig. 2. Heatmap coefficient matrix between samples of *P. fulvidraco* and *O. niloticus*. Page 3

Supplementary Table 1. Primer sequences used for qRT-PCR. Page 4-5

Supplementary Table 2. Sequencing data processing results of four fish species. Page 6-7

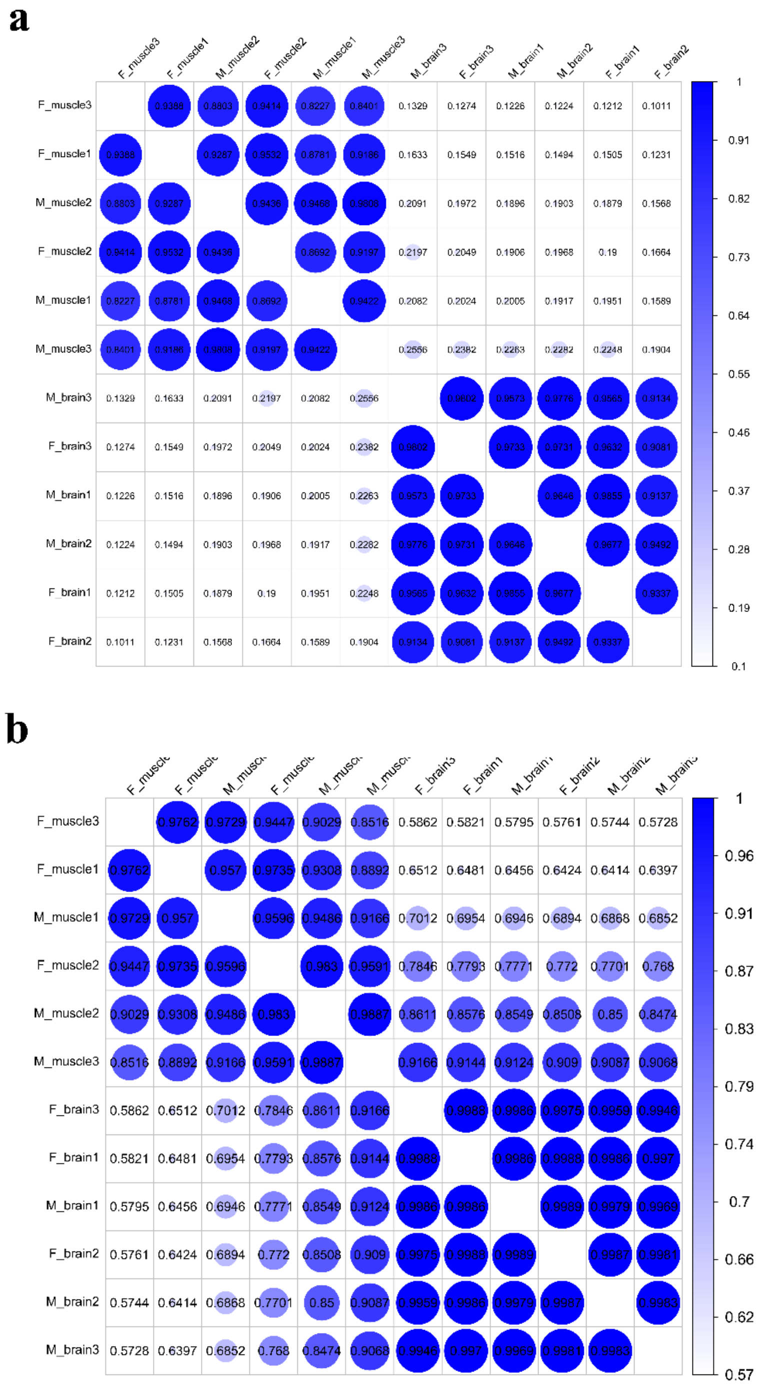

Supplementary Fig. 1. Heatmap coefficient matrix between samples of *M. anguillicaudatus* and *C. semilaevis*. (a) Heatmap coefficient matrix of *M. anguillicaudatus*. (b) Heatmap coefficient matrix of *C. semilaevis*. Both of the abscissa and ordinate represent the sample names, and the color represents the value of correlation coefficient, the darker the color, the closer it is to 1, indicating a higher similarity of expression patterns between samples.

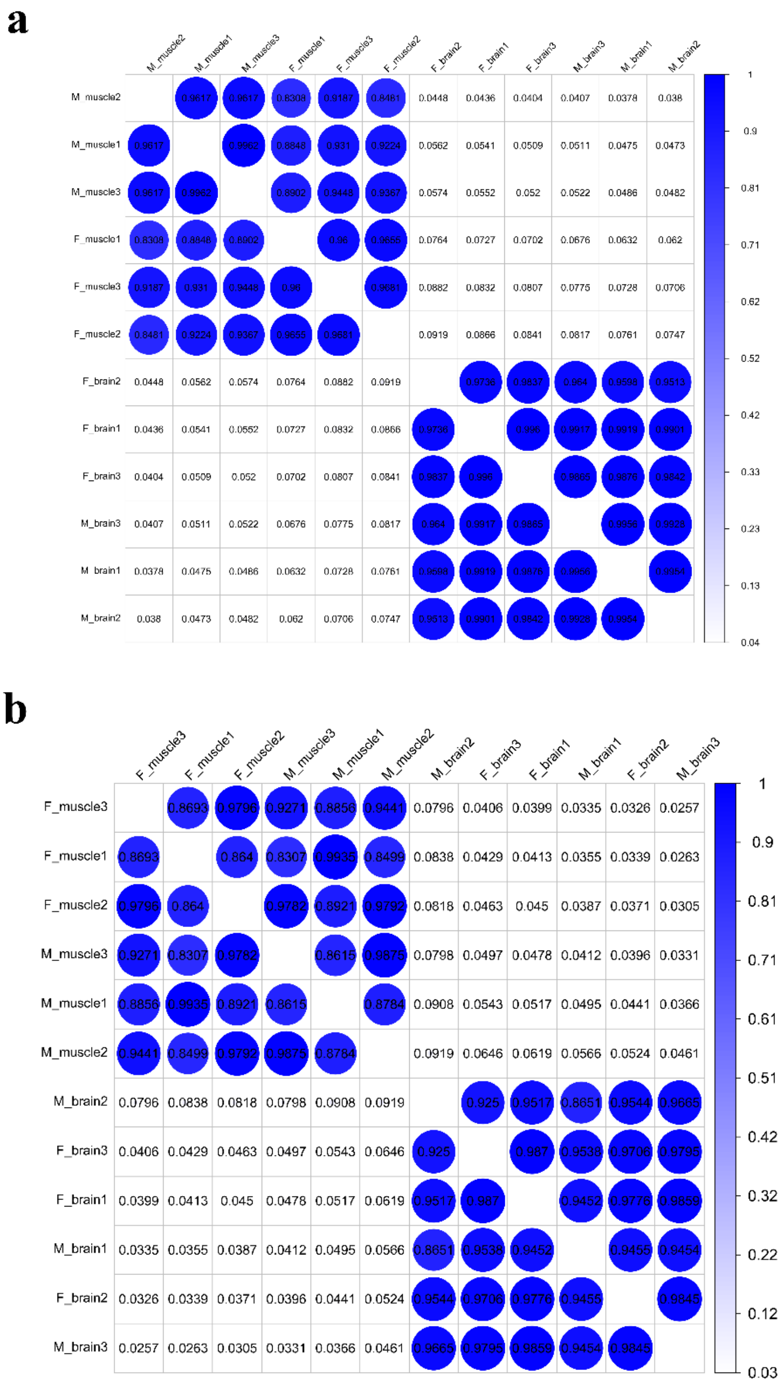

Supplementary Fig. 2. Heatmap coefficient matrix between samples of *P. fulvidraco* and *O. niloticus*. (a) Heatmap coefficient matrix of *P. fulvidraco*. (b) Heatmap coefficient matrix of *O. niloticus*. Both of the abscissa and ordinate represent the sample names, and the color represents the value of correlation coefficient, the darker the color, the closer it is to 1, indicating a higher similarity of expression patterns between samples.

Supplementary Table 1. Primer sequences used for qRT-PCR.

Primer name	Sequences (5’→3’)	Application
Magh-qF	AGAGGCTCCCACTGGCAAAG	Used for quantification in *M. anguillicaudatus*
Magh-qR	GCACGCTGATGCCCACTTT	
Macd22-qF	TTCTACAGTAAGTTACTGGCAACCA	
Macd22-qR	AAGTTCAGGGTCAAGCACACA	
Masqle-qF	CGGACCGGATAGTGGGG	
Masqle-qR	CAGCACGGCGCAGTCC	
Maprl-qF	GTTTGAACAGTATTTCCTGCCCA	
Maprl-qR	GGTGTAACTGGAACAAAGGGTCC	
MaTnnT3-qF	AGATCCCTGATGGAGAGAAAGTG	
MaTnnT3-qR	CTTGTCCTTCTCAGCACGGA	
Mamyplf-qF	GGTTCCTCCAATGTTTTCTCCA	
Mamyplf-qR	AACGGTGAAGTTGATTGGGC	
Maerbb3-qF	TAAGCGTTTTGTATCGGAGGAGT	
Maerbb3-qR	GGTATCCACACTCCCTTATGCAC	
Mahsp70-qF	AGCTGAGCGTGCAGTGTTGT	
Mahsp70-qR	GGAGTAGGTGGTGCCCAGGT	
Maβ-actin-qF	CTCAATCCCAAAGCCAACAG	
Maβ-actin-qR	GGAAGAGCATAACCCTCGTAGA	
Csgh-qF	CAGAATCAGAACCAAACCAAGC	Used for quantification in *C. semilaevis*
Csgh-qR	TGAATTTTCAAAGTCGGAGAAGTAT	
Cscd22-qF	GTCAGACTGTAAAGAATGTGGCG	
Cscd22-qR	CCCCAGCATCTCTGTTTTCTACT	
Cssqle-qF	ATAAGAAGTCTGATTCCGTTTGGAA	
Cssqle-qR	ACCTGTTTGGGATTAAATGTTTGA	
Csprl-qF	ACAAAATCCACTCCCTCAGCA	
Csprl-qR	AGCAGGTCCGACTCAGACACTT	
CsTnnT3-qF	TCAGAATAAGGACCTGGTTGAGC	
CsTnnT3-qR	TCTCTTTGTCTTTCTCAGCACGA	
Csmyplf-qF	CAACCGAACCTCCACTCATACC	
Csmyplf-qR	CCTAAGGTCGTCTTTGCTGATGAT	
Cserbb3-qF	ACGACGTCAGAAGCTAATTGAAAT	
Cserbb3-qR	ATTGTGAAGCAAACTCACGCC	
Cshsp70-qF	GCAGGAGAGTGCCCGTTACC	
Cshsp70-qR	CTCGTCAAACTCCGCAGGCA	
Csβ-actin-qF	GTAGGTGATGAAGCCCAGAGCA	
Csβ-actin-qR	CTGGGTCATCTTCTCCCTGT	
Pfgh-qF	AGTGTTGCTCTCTGTGGTGTTGG	Used for quantification in *P. fulvidraco*
Pfgh-qR	TTGCAGAAACTCAGGGGGAAG	
Pfcd22-qF	CCTCTCCACATCCTACACTGGAGT	

Pfcd22-qR	TCGGAGGTTTGAGGGATTAGG	
Pfsqle-qF	AGAGCTGGTGCTGGCAAACC	
Pfsqle-qR	GGCAGGCATCGTCCTGAGAC	
Pfprl-qF	GCGACCTGCACTCAGAGCTT	
Pfprl-qR	CTCGTGCAGCAGGGACAGAG	
PfTnnT3-qF	AAGCCAAAGTTCAGACCCAGTG	
PfTnnT3-qR	CTGCTCTGCTCTCTCAGCCCTA	
Pfmyplf-qF	CGGCTTCACACAGTCTTCTCTTC	
Pfmyplf-qR	CCGTCTCTGTTCTGGTCGATG	
Pferbb3-qF	CGAACTGCACAAGCGGATGC	
Pferbb3-qR	GCGTCGGATGGACAGGCTAC	
Pfhsp70-qF	CAAGTGGCTATGAACCCCAA	
Pfhsp70-qR	CCAAGATAAGCCTCTGCGATT	
Pfβ-actin-qF	CCTGAGAAACGGCTACCACATCC	
Pfβ-actin-qR	AGCAACTTTAATATACGCTATTGGAG	
Ongh-qF	CTGTCGGTTGTGTGTTTGGG	
Ongh-qR	CAGGAAGATTTTGTTGAGCTGAC	
Oncd22-qF	ACTGTCAAGGTTATTTATGCCCC	
Oncd22-qR	CACGACATGTGTAATCACCGCT	
Onsqle-qF	AGACCTCGCGATGGTTGCAG	
Onsqle-qR	TTCTCGCTCCTCGGTGTTGC	
Onprl-qF	TCCCTCCTCCAAGCCTGGTC	
Onprl-qR	CCTCCTCGGTAGGGCAGTGA	
OnTnnT3-qF	GAGAGGATTGAGAAGCGTCGTG	Used for quantification in *O. niloticus*
OnTnnT3-qR	CATGCTGGACAGAGCCGACT	
Onmyplf-qF	GTCACATCGGCTTGGCGTAG	
Onmyplf-qR	TCGTCCTTGCTGATGATGCC	
Onerbb3-qF	TGACGGACCACACCAGAGACT	
Onerbb3-qR	AGGCCATGCTCTCCATCCCT	
Onhsp70-qF	AGGCAAAACAGGAGAACGC	
Onhsp70-qR	ATTCTGGCTAATGTCCTTCTTATGT	
Onβ-actin-qF	GGTGGGTATGGGTCAGAAAGA	
Onβ-actin-qR	GCTGTCGTGAAGGAGTAG	

Supplementary Table 2. Sequencing data processing results of four fish species.

Species	Sample	Raw reads	Clean reads	Mapped reads
M. anguillicaudatus	F_brain1	46,853,242	46,063,672	40,823,402(88.62%)
	F_brain2	55,042,498	54,078,494	47,824,036(88.43%)
	F_brain3	55,897,582	54,717,918	48,806,088(89.20%)
	F_muscle1	50,938,706	50,031,922	42,887,904(88.58%)
	F_muscle2	50,268,054	49,482,766	42,807,721(88.99%)
	F_muscle3	48,313,080	47,547,164	46,219,756(89.18%)
	M_brain1	49,417,990	48,417,470	47,699,445(95.34%)
	M_brain2	48,985,084	48,104,112	47,398,182(95.79%)
	M_brain3	52,732,672	51,828,214	45,714,030(96.14%)
	M_muscle1	49,118,220	48,267,984	46,117,857(95.55%)
	M_muscle2	46,481,320	45,718,052	43,555,226(95.27%)
	M_muscle3	55,342,976	54,471,750	51,901,596(95.28%)
C. semilaevis	F_brain1	52,674,560	50,575,360	46,356,321(93.87%)
	F_brain2	56,084,480	53,288,960	48,668,788(93.52%)
	F_brain3	51,189,760	49,121,280	44,935,026(93.68%)
	F_muscle1	51,118,080	49,213,440	45,098,826(93.83%)
	F_muscle2	59,095,040	56,596,480	52,198,147(94.43%)
	F_muscle3	55,500,800	53,360,640	49,281,040(94.57%)
	M_brain1	52,992,000	50,964,480	46,759,700(93.95%)
	M_brain2	56,422,400	52,633,600	48,278,429(93.92%)
	M_brain3	55,347,200	52,561,920	48,361,155(94.21%)
	M_muscle1	59,688,960	57,354,240	52,875,749(94.40%)
	M_muscle2	49,039,360	46,315,520	42,514,050(94.00%)
	M_muscle3	60,661,760	57,221,120	52,436,630(93.84%)
P. fulvidraco	F_brain1	51,312,640	50,186,240	46,693,209(95.28%)
	F_brain2	54,353,920	52,971,520	49,105,905(94.92%)
	F_brain3	52,551,680	51,240,960	47,434,420(94.80%)
	F_muscle1	56,883,200	55,797,760	52,680,033(96.67%)
	F_muscle2	52,531,200	51,527,680	48,554,205(96.50%)
	F_muscle3	54,138,880	53,084,160	50,035,466(96.53%)
	M_brain1	58,368,000	57,128,960	53,046,399(95.08%)
	M_brain2	57,538,560	56,217,600	52,102,848(94.90%)
	M_brain3	53,678,080	52,500,480	48,761,169(95.10%)
	M_muscle1	58,746,880	57,733,120	54,127,023(96.00%)
	M_muscle2	57,098,240	56,084,480	52,862,630(96.52%)
	M_muscle3	55,255,040	54,220,800	51,329,225(96.95%)
O. niloticus	F_brain1	53,288,960	52,111,360	47,684,210(93.70%)

	F_brain2	50,810,880	49,643,520	45,855,855(94.59%)
	F_brain3	55,162,880	53,821,440	48,724,394(92.70%)
	F_muscle1	52,551,680	51,189,760	48,515,924(97.05%)
	F_muscle2	50,944,000	49,827,840	47,653,410(97.93%)
	F_muscle3	47,646,720	46,714,880	44,043,554(96.55%)
	M_brain1	54,138,880	52,858,880	48,604,242(94.16%)
	M_brain2	54,333,440	52,858,880	47,922,342(92.84%)
	M_brain3	51,824,640	50,513,920	47,346,278(95.98%)
	M_muscle1	52,439,040	51,425,280	47,943,278(95.46%)
	M_muscle2	55,592,960	54,302,720	51,067,715(96.30%)
	M_muscle3	55,674,880	54,425,600	52,024,305(97.89%)
